# Supplementary material for: Analysis of PPARγ Signaling Activity in Psoriasis
Source: Int J Mol Sci. 2021 Aug 10;22(16):8603. doi: 10.3390/ijms22168603 (PMC8395241; doi:10.3390/ijms22168603)
Supplement: Supplementary file 1 [file ijms-22-08603-s001.zip › Supplemental materials_Analysis of PPARg signaling activity in psoriasis/Pathway models/Models images and html files/Model of PPARG related pathways in psoriasis (short version)/261.html]

ADCY


# Functional Class ADCY

|  |  |
| --- | --- |
| URN | urn:agi-aopfc:0000480 |
| Total Entities | 10 |
| Connectivity | 5170 |
| Name | ADCY |
| Description | Adenylate cyclase |

---

|  |  |
| --- | --- |
| ChildConcepts | ADCY1 |
|  | ADCY2 |
|  | ADCY3 |
|  | ADCY4 |
|  | ADCY5 |
|  | ADCY6 |
|  | ADCY7 |
|  | ADCY8 |
|  | ADCY9 |
|  | ADCY10 |

---

|  |  |
| --- | --- |
| Pathway | Circadian Clock Genes Mutations Cause Insomnia |
|  | Melatonin Effects on Circadian Cycle |
|  | Hypoxia Inhibits mTOR Signaling |
|  | High Fat Diet Activates cAMP Related Exocytosis in Synapse and Endocrine Cell |
|  | GCG and PPY Regulate Metabolism and Satiety |
|  | Ghrelin and Nesfatin-1 Regulate Stomach Functional State and Satiety |
|  | mTOR Signaling Activation by Taste Sensor Receptors (Rodent Model) |
|  | Ghrelin and SST Regulate GH1 Release in Hypothalamus |
|  | L-cell: GCG, PYY and 5-HT Release |
|  | P-cell Activation |
|  | K-cell: GIP Production |
|  | I-cell: CCK-8 Secretion and Regulation of Eating Behavior |
|  | D-cell: Somatostatin Release |
|  | M-cell: Motilin Release |
|  | A-cell (X-like): Ghrelin and Nesfatin-1 Synthesis |
|  | G-cell: Gastrin Secretion |
|  | ECL-cell: CHGA and Histamine Synthesis |
|  | N-cell: Neurotensin Production |
|  | Insulin Synthesis in beta-Cell |
|  | Insulin Release Inhibition by Somatostatins |
|  | Dopamine Receptors Signaling |
|  | Serotonin Receptors Signaling |
|  | Catecholamines Secretion from Adrenal Gland |
|  | Circadian Clock and Sleep Regulation by Melatonin |
|  | Norepinephrine Release Regulation |
|  | Dopamine Mediated Glutamate Release/Uptake Circle in Neuron in Migraine |
|  | Dopamine Synthesis and Release |
|  | CRH Secretion Regulation |
|  | Prolactin Production |
|  | Somatostatin Receptors Signaling |
|  | POMC Secretion in adenohypophysis |
|  | Testicular AMH Production |
|  | LH and FSH Signaling and Steroidogenesis |
|  | Estrogens in Prolactin Production |
|  | Steroid Hormones Steroidogenesis |
|  | CXC Chemokine Receptor Signaling |
|  | CC Chemokine Receptor Signaling |
|  | Proplatelet Maturation |
|  | Hypothalamic-Pituitary-Adrenal Axis in Acute Phase Response |
|  | MC1R in anti-Inflammatory Signaling |
|  | Prostaglandin E2 Receptor Signaling in Neurons |
|  | Leukotriene Effect on Vascular Endothelial Cell Response |
|  | Eicosanoids in Myeloid Cell Activation |
|  | Histamine Receptor H3 Signaling in Neurons |
|  | Prostaglandin E2 and Histamine in Gastric Acid Secretion |
|  | Neutrophil Activation via LTB4/Polyisoprenyl Phosphate Signaling |
|  | Basophil Activation |
|  | Mast-Cell Activation without Degranulation through CRHR1 Signaling |
|  | Mast-Cell Activation without Degranulation |
|  | Mast-Cells De Novo Synthesized Mediators via IgE Independent Signaling |
|  | Neutrophil Chemotaxis |
|  | SNS Related Adrenal Cortex Response |
|  | Vascular Endothelial Cell Activation by NO |
|  | Vasodilation Activation |
|  | Vascular Endothelial Cell Activation by Cytokines |
|  | Lipolysis Regulation in Adipocyte |
|  | Synaptic Potentiation by PKMZ in LTP Maintenance - Active State |
|  | Potassium Flux Regulation |
|  | Nociception Expression Targets Signaling |
|  | Sodium Flux Regulation |
|  | Vascular Motility |
|  | Ca2+ Flux Regulation |
|  | TAAR1 -> Neurotransmitter Uptake |
|  | NPY1R -> CRH/POMC Production |
|  | CGRP -> Calcium Influx |
|  | CRH -> Synthesis of Corticosteroids |
|  | EDNRA/B -> Vascular Motility |
|  | ADORA2A/B -> Vasodilation |
|  | ADORA1/2A -> Exocytosis |
|  | ADRB1/3 -> Vasodilation |
|  | ADRA1 -> Ion Channels |
|  | ADRA2C/ADRB2 -> Vasoconstriction |
|  | ADRB1 -> Ion Channels |
|  | ADRA2C/ADRB2 -> Synaptic Endocytosis |
|  | ADRA2A -> Hyperpolarization |
|  | ADRB1 -> Prostaglandin Generation |
|  | BDKRB1/2 -> Vasodilation |
|  | BDKRB1/2 -> Ion Channels |
|  | CNR1/2 -> Membrane Transport |
|  | CNR1/2 -> Vascular Motility |
|  | CNR1/2 -> IL1B/2/4/6/10 Production |
|  | DRD1/5 Expression Targets |
|  | DRD2/4 -> Membrane Transport |
|  | GALR1/2/3 -> POMC/NPY Production |
|  | GALR1/2/3 -> Neurotransmitter Metabolism |
|  | HRH1/2 -> Membrane Polarization |
|  | HRH1/3 -> Synaptic Transmission |
|  | HRH1/2 -> Vascular Motility |
|  | HRH2/4 -> IL6/10 Production |
|  | CHRM1/2/3/5 -> Ion Channels |
|  | CHRM1/2/3 -> Vascular Motility |
|  | OPRD/OPRM -> Ion Channels |
|  | OPRK -> Pain Perception |
|  | OPRL1 -> Ion Channels |
|  | PTGIR -> IL6 Production |
|  | PTGDR -> Vasodilation |
|  | PTGER1/4 -> Vascular Motility |
|  | PTGER2/3 -> Vascular Motility |
|  | PTGIR -> Vasodilation |
|  | HTR4/6/7 -> Vasodilation |
|  | HTR7 -> IL6 Production |
|  | HTR4/6/7 -> Cation Channels |
|  | HTR2 -> Membrane Transport |
|  | HTR1 -> Vascular Motility |
|  | TACR1 -> Membrane Transport |
|  | GABA(B)R -> Postsynaptic Inhibition |
|  | GRM2-4/6-8 (Presynaptic) -> Glutamate Release Attenuation |
|  | GRM1/5 (Postsynaptic) -> Ion Channels |
|  | P2RY11/13/14 -> IL8/10 Production |
|  | GPR3 in Oocyte Meiotic Arrest |
|  | Agomelatine Antidepressant Action |
|  | Histamine in Arousal Regulation |
|  | Endocannabinoids Role in Sleep Regulation |
|  | Circadian Clock Genes in Suprachiasmatic Nuclei Neurons |
|  | Prostaglandins Role in Sleep Regulation |
|  | HRH3 in Negative Regulation of Histamine Production and Release in Sleep Regulation |
|  | Adenosine Role in Sleep Regulation |
|  | Retinal Ganglion and Suprachiasmatic Nucleus in Melatonin Synthesis Regulation |
|  | Melatonin Synthesis in Pinealocytes |
|  | non-Genomic Rapid Actions of Vitamin D in Vitamin D Biology |
|  | Gap Junction Assembly |
|  | beta-Cell Granules Exocytois |
|  | Cardiomyocyte Dysfunction |
|  | Dilated Cardiomyopathy |
|  | Alcoholic Dilated Cardiomyopathy (Mouse Model) |
|  | Ca2+ Signaling in Dilated Cardiomyopathy |
|  | Angiotensin-Aldosterone System Activation Scheme |
|  | Arterial Hypertension |
|  | Endothelial Cell Dysfunction in Pulmonary Hypertension |
|  | Vascular Reactivity in Raynaud Disease |
|  | NO Synthesis in Early Vasospasm |
|  | Contraction Due Vasospasm |
|  | Sebocyte Proliferation in Acne Vulgaris |
|  | Hyperseborrhea in Acne Vulgaris |
|  | Melanoma |
|  | MITF as a Regulator of Melanoma Cell Development |
|  | Melanocyte Role in Skin Aging |
|  | Wolfram Syndrome Progression (Hypothesis) |
|  | Leptin in Insulin Synthesis and Secretion |
|  | beta-Cell Destruction through Cytokines in Diabetes Mellitus |
|  | beta-Cell Mass Regulation |
|  | beta-Cell Recovery in Diabetes Mellitus |
|  | G-Proteins Signaling in Insulin Secretion in beta-Cell |
|  | beta-Cell Function Inhibition by Ciclosporine and Tacrolimus (Rodent Model) |
|  | Ghrelin Influence on Insulin Secretion (Hypothesis) |
|  | Ca2+ Reabsorption Decline in Intestine |
|  | Ca2+/Pi Reabsorption Decline in Kidney in Hyperparathyroidism |
|  | Hypothyrodism Associated Hyperprolactinemia |
|  | DRD2 Pharmacological Inhibition in Hyperprolactinemia |
|  | Ethanol Induced Efects in Hyperprolactinemia |
|  | Cardiovascular Effects in Hyperthyroidism |
|  | GNRH Signaling Impairment in Hypogonadotrophic Hypogonadism |
|  | Ca2+/Pi Reabsorption Decline in Kidney in Hypoparathyroidism |
|  | Osteoblast Dysfunction in Hypoparathyroidism |
|  | Thyroid Stimulating Hormone (TSH) Secretion in Overt Hypothyroidism |
|  | Central Hypothyroidism |
|  | Tertiary Hypothyroidism |
|  | Prolactinoma Overiew |
|  | Metastatic Colorectal Cancer |
|  | Basophil Activation in Asthma |
|  | CFTR Facilitates Oxidative Stress in Airway Epithelium in CF |
|  | Airway Epithelial Cell Dysfunction |
|  | CFTR Expression in Epithelial Cells (Class I Mutations) |
|  | CFTR Related Ion-Channel Dysfunction in Cystic Fibrosis Airway Epithelium (Class III Mutations) |
|  | Glutamate Toxiicity Effects on Striatum Medium Spiny Neurons in Hantington's Disease |
|  | Proteins Involved in Epilepsy |
|  | ADK Expression Downregulation after Acute Seizures |
|  | Apoptosis and Immediate Early Gene Activation in Epileptiform Disorders |
|  | Vascular Endothelial Cell Vasodilation in Brain |
|  | Nitroxyl and CGRP in Migraine Pain Activation |
|  | Dopamine Synthesis and Release in Migraine |
|  | Dopamine Mediated Glutamate Release and Glutamate Uptake Circle |
|  | Vascular Smooth Muscle Cell Vasodilation in Brain |
|  | eNOS (NOS3) Activation via Prostaglandin E2 and Histamine |
|  | NO Effect on Vasodilation and Vasoconstriction |
|  | GABRB3 Mutation and Adenosine Deaminase Polymorphism |
|  | Sleep Disturbance (Hypothesis) |
|  | Prostaglandin D2 Synthase Downregulation |
|  | Local Estrogen Production in Endometriosis |
|  | LH and FSH Signaling Impairment in Primary Ovarian Insufficiency |
|  | MIF Role in Cholesteatoma |
|  | Cholesteatoma |
|  | HPV E5 Regulates EDNRA and EGFR Signaling Pathway in Keratinocytes |
|  | Noradrenalin Signaling in Glaucoma |
|  | Serotonin Signaling in the Ciliary Body |
|  | Cochlear Hair Cell Synapse Proteins Mutations (Age-Related/Congenital) |
|  | Polycystins Mutation Effects in Cystic Kidney Disease |
|  | Penile Detumescense Augmentation Mechanisms |
|  | Penile Erection Impairment |
|  | Cryptorchidism Molecular Mechanism (Hypothesis) |
|  | Sertoli Cells Dysfunction Causes Male Infertility |
|  | Ions Reabsorption Dysregulation in Nephrolithiasis |
|  | Ca2+ Reabsorption Dysregulation in Urolithiasis |
|  | Noradrenaline/Gs Expression Targets |
|  | Dopamine/Gs Expression Targets |
|  | Serotonin/Gs Expression Targets |
|  | Epinephrine/Gs Expression Targets |
|  | GLP1R -> ARRB1 Signaling |
|  | AGTR1 -> ARRB1/ARRB2 Signaling |
|  | GPCRs Desentization |
|  | Hedgehog Family -> ARRB1/ARRB2 Canonical Signaling |
|  | CXCL6 Expression Targets |
|  | CXCL1 Expression Targets |
|  | CXCL2 Expression Targets |
|  | CXCL3 Expression Targets |
|  | CXCL5 Expression Targets |
|  | Complement Component GPCRs Signaling |
|  | Valdecoxib Induced Ischemic Disease |
|  | Doxorubicin Induced Cardiotoxicity |
|  | GCG Expression Targets |
|  | ADCYAP1 Expression Targets |
|  | FSHR Expression Targets |
|  | Glutamate/Gq Expression Targets |
|  | VIP Expression Targets |
|  | HIF1A in Vasculogenic Mimicry of Cancer |
|  | Myeloid Derived Suppressor Cells in Cancer Immune Escape |
|  | Adenosine/cAMP Promote Immunosuppression by Treg Cells |
|  | HCAR1/HCAR2 -> MAPK Signaling |
|  | Thromboxane A2 Expression Targets |
|  | PAF/Gq -> AP-1/ATF1/CREB/ERK/SRF Expression Targets |
|  | Dronabinol/Anandamide Expression Targets |
|  | S1P Expression Targets |
|  | PGE1 Expression Targets |
|  | MTNR1 Signaling |
|  | Purine Metabolism |
|  | OR1A1 -> GNAS Signaling |
|  | AVP/Gs -> CREB/ELK/SRF/AP-1/EGR Expression Targets |
|  | AGT -> TP53 Expression Targets |
|  | EDN1 Expression Targets |
|  | AGT -> CREB Expression Targets |
|  | SST Expression Targets |
|  | NPY Expression Targets |
|  | AGT -> ELK/SRF Expression Targets |
|  | TAC1 Expression Targets |
|  | EDN3 Expression Targets |
|  | Lysophosphatidic Acid/LPARs Signaling |
|  | Estrogens/ESR1 non-Genomic Signaling |
|  | ERK/MAPK Canonical Signaling |
|  | Hedgehog Family Signaling |
|  | IL1B -> PGE2 Expression Targets |
|  | ROS and RNS in Vasoconstriction and Vasodilation Regulation |
|  | Model of PPARG signaling in psoriasis |
|  | Model of PPARG related pathways in psoriasis (short version) |
|  | 3\_Increased proliferation of sebocytes in acne vulgaris |
|  | 2\_Hyperseborrhea in acne vulgaris |
|  | 1\_Osteoclast-mediated bone resorption in postmenopausal women |
|  | 1\_Formation of cysts in polycystic kidney disease |
|  | 1\_Local estrogen production stimulates ectopic endometrium proliferation |
|  | 2\_Impaired steroidogenesis in PCOS |
|  | 2\_Dysfunctions of cochlear hair cell synapse proteins in hearing loss |
|  | 1\_Endothelial cells stimulate vasoconstriction and thrombosis in pulmonary hypertension |
|  | 3\_Cardiomyocyte dysfunction unrelated to cell junctions in ARVD |
|  | 4\_Endothelial cells repress vasoconstriction in hypertension |
|  | 3\_1\_Mucus accumulation in asthma |
|  | 4\_2\_Airway smooth muscle cells proliferation and airway tissue remodeling |
|  | 4\_Mucin hyperproduction in goblet and mucous cells in COPD |
|  | 1\_Intestine epithelial cell dysfunction in ulcerative colitis |
|  | 3\_1\_Beta-cell proliferation and compensation: proliferation |
|  | 1\_2\_Insulin synthesis and secretion: insulin secretion |
|  | 1\_1\_Insulin synthesis and secretion: insulin processing and storage |
|  | 3\_2\_Beta-cell proliferation and compensation: neogenesis/dedifferentiation |
|  | 1\_CFTR expression and degradation in epithelial cells in cystic fibrosis (class I-II mutations) |
|  | 3\_Pain perception in diabetic neuropathy |
|  | 1\_2\_Glutamate overload and NO-mediated vasodilation |
|  | 1\_Decreased secretion of thyroid hormones |
|  | Model of PPARG signaling in psoriais (tested) |

---

|  |  |
| --- | --- |
| MedScan ID | 12813770 |

---

|  |  |
| --- | --- |
| Alias | adenylcyclase systems |
|  | adenyl cyclase |
|  | adenylate cyclases |
|  | 4.6.1.1 |
|  | ADCYs |
|  | ATP pyrophosphate-lyases |
|  | 3',5' adenylate synthetase |
|  | adenylylcyclase |
|  | ATP pyrophosphate-lyase |
|  | adenylate cyclase |
|  | atp pyrophosphate lyase (cyclizing) |
|  | adenylylcyclases |
|  | adenyl cyclases |
|  | 3',5' cyclic amp synthetase |
|  | ADCY |
|  | adenylyl cyclase |
|  | cAMP generating peptide |
|  | adenylcyclase system |
|  | ATP diphosphate-lyase (cyclizing) |
|  | adenylyl cyclases |
|  | adenylcyclase |
|  | 3',5'-cyclic AMP synthases |
|  | 3',5'-cyclic AMP synthetases |
|  | cAMP generating peptides |
|  | ATP diphosphate-lyase (cyclizing; 3',5'-cyclic-AMP-forming) |
|  | ATP diphosphate-lyases (cyclizing) |
|  | 3',5'-cyclic AMP synthetase |
|  | adenylcyclases |
|  | 3',5'-cyclic AMP synthase |

---

|  |  |
| --- | --- |
| EC Number | 4.6.1.1 |

---

|  |  |
| --- | --- |
| Cell Localization | Cytoplasm |

---
